# Predictive factors of the accelerated transepithelial corneal cross-linking outcomes in keratoconus

**DOI:** 10.1186/s12886-021-02235-4

**Published:** 2022-01-03

**Authors:** Mi Tian, Weijun Jian, Xiaoyu Zhang, Ling Sun, Yang Shen, Xingtao Zhou

**Affiliations:** 1 Eye Institute and Department of Ophthalmology, Eye and ENT Hospital, Fudan University, No. 19 Baoqing Road, Shanghai, 200031 China; 2 NHC Key Laboratory of Myopia (Fudan University); Key Laboratory of Myopia, Chinese Academy of Medical Sciences, Shanghai, China; 3 Shanghai Research Center of Ophthalmology and Optometry, Shanghai, China

**Keywords:** Keratoconus, Accelerated transepithelial corneal cross-linking, Prospective study, Corneal thickness, Keratometry

## Abstract

**Background:**

This study aimed to evaluate the clinical outcomes and assess preoperative characteristics that may predict outcomes in keratoconus 1 year after accelerated transepithelial corneal cross-linking (ATE-CXL).

**Methods:**

This prospective study included 93 eyes of 84 consecutive keratoconus patients with 1-year follow-up after ATE-CXL. Preoperative characteristics included corneal astigmatism, anterior chamber depth, anterior chamber volume, radius of curvature, posterior elevation, central corneal thickness (CCT), thinnest corneal thickness, steepest meridian keratometry, flattest meridian keratometry, and the maximum keratometry (Kmax). Data were obtained preoperatively and at 1, 3, 6, and 12 months postoperatively. The patient eyes were grouped into 3 subgroups according to CCT and Kmax values to observe the changes of keratoconus progression.

**Results:**

All patients were successfully operated without complications at any follow-up time point. Mean changes of Kmax from baseline at 6 and 12 months were − 0.60 ± 2.21 D (*P* = 0.011) and − 0.36 ± 1.58 D (*P* = 0.030), respectively. Eyes with a thinner CCT and higher Kmax values exhibited a tendency for topographic flattening of ≥1.0 D (*P* = 0.003; P = 0.003). In the subgroup comparison, the Kmax values decreased significantly at 6 and 12 months after ATE-CXL in the group with CCT ≤ 450 μm (*P* = 0.018 and *P* = 0.045); the Kmax values of the group with Kmax > 65.0 D decreased significantly at 6 months postoperatively (*P* = 0.025).

**Conclusion:**

ATE-CXL is a safe and effective treatment for keratoconus patients. Patients with thinner CCT and higher Kmax values are more likely to benefit from ATE-CXL.

## Background

Keratoconus is a progressive degeneration disease, in which the cornea becomes thinner, leading to irregular astigmatism and irreversible loss of vision [1]. Corneal cross-linking (CXL) is one of the treatments aimed at stabilizing the progression of keratoconus [2–4]. During the CXL procedure, a photochemical reaction is initiated to create extra or new chemical chains in the corneal stroma [5]. CXL enhances corneal hardness by increasing the covalent binding between collagen fibers [6–8]. Previously, studies have reported the efficacy of CXL in the visual and morphological improvement of keratoconus [9–14]. Accelerated transepithelial corneal cross-linking (ATE-CXL) is a treatment based on CXL, which can preserve the integrity of the cornea without removing the corneal epithelium and Bowman’s layer (epithelium-on). It can also increase patients’ cooperation by shortening the time of riboflavin infiltration and ultraviolet radiation. Our team has reported on the long-term safety and efficacy of ATE-CXL as a treatment for adolescent and adult keratoconus [15–18]. However, the efficacy of CXL may vary among patients. Therefore, more reports need to be published to help predict the postoperative results; this will help clinicians choose the treatment and gauge complications. Thus, the following question arises: Can preoperative factors be used to predict the possibility of stabilizing progression or improving corneal morphology after CXL?

Previous studies have reported how preoperative factors, including maximum keratometry (Kmax), central corneal thickness (CCT), thinnest corneal thickness (TCT) and the cone position affected the clinical outcomes of conventional corneal cross-linking (C-CXL; epithelium-off) [19–23] and accelerated corneal cross-linking (A-CXL; epithelium-off) [24, 25]. In the recent study [17], Zhang found that patients with thinner TCT showed more decreasing in average keratometry after ATE-CXL. However, the relationship between preoperative factors and postoperative outcomes of ATE-CXL are yet to be elucidated. Here, we evaluated the topographical outcomes and analyzed the preoperative parameters that may impact the corneal morphological changes of keratoconus patients up to 1 year after ATE-CXL.

## Patients and methods

### Patients

The collected data consisted of 93 eyes (44 right eyes and 49 left eyes) from 84 patients (60 male and 24 female, mean age: 24.51 ± 4.99 years) with progressive keratoconus diagnosed at the Eye and ENT Hospital of Fudan University in Shanghai, China. All recruited patients were treated by ATE-CXL (registration number: ChiCTR-OIC-16008181 29/03/2016). This study was approved by the Ethics Committee of the Eye and ENT Hospital of Fudan University and adhered to the tenets of the Declaration of Helsinki. Informed consent was obtained from all patients after a detailed explanation of the procedure prior to treatment.

The following features were defined as inclusion criteria for the study: 1) age over 18 years, 2) progressive keratoconus, defined as an increase in Kmax values, spherical refractive equivalent or astigmatism of > 1 D in 1 year, 3) the TCT > 380 μm, 4) no ophthalmological disease affecting the cornea. Exclusion criteria were as follows: 1) worn rigid gas permeable lenses and soft contact lenses of more than 4 and 2 weeks, respectively, 2) stromal scarring in the cornea, 3) allergy to any eye drops, 4) an ocular surgical history. The baseline characteristics are presented in Table 1.

**Table 1:** Demographics and characteristics of patients (Mean ± SD)

	Mean ± SD	Range
Age (year)	24.51 ± 4.99	(18, 37)
Corneal astigmatism (D)	3.81 ± 2.58	(0.1, 12.2)
ACD (mm)	3.40 ± 0.27	(2.76, 4.08)
ACV (μL)	205.09 ± 27.66	(133, 269)
K1 (D)	48.65 ± 5.44	(39.2, 65.1)
K2 (D)	52.47 ± 6.52	(39.6, 71.6)
Kmax (D)	60.25 ± 10.10	(40, 91.4)
CCT (μm)	473.75 ± 41.34	(386, 589)
TCT (μm)	455.37 ± 43.55	(381, 579)

*ACD* anterior chamber depth, *ACV* anterior chamber volume, *K1* steepest meridian keratometry, *K2* flattest meridian keratometry, *Kmax* maximum keratometry, *CCT* central corneal thickness, *TCT* thinnest corneal thickness

### Grouping and result judgment criteria

Patients were divided into three groups based on their CCT and the Kmax values preoperatively.

1) According to CCT: Group 1, CCT > 500 μm; group 2, CCT 450 to 500 μm; and, group 3, CCT < 450 μm.

2) According to Kmax: Group 1, Kmax values > 65 D; group 2, Kmax values 55 to 65 D; and, group 3, Kmax values < 55 D.

Keratoconus progression was defined as an increase of > 1.0 D in Kmax values, stabilization was defined as a change between − 1.0 D and + 1.0 D, and regression was defined as a decrease of > 1.0 D.

### Measurements

All patients underwent slitlamp biomicroscopy examination, endothelial cell density (ECD) and best corrected visual acuity (BCVA) assessments preoperatively and postoperatively. Anterior segment tomography parameters obtained by Pentacam (OCULUS Optikgeräte GmbH; Wetzlar, Germany), including corneal astigmatism, anterior chamber depth (ACD), anterior chamber volume (ACV), CCT, TCT, steepest meridian keratometry (K1), flattest meridian keratometry (K2), Kmax, anterior radius of curvature (ARC), posterior radius of curvature (PRC), posterior central elevation (PCE), posterior mean elevation (PME), were recorded preoperatively and at 1, 3, 6, and 12 months post operation. The ARC and PRC taken from a 3.0-mm optical zone centered on the thinnest point. The PME was calculated as the mean of 27 points in the central 4.0 mm zone of the posterior corneal surface.

### Surgical procedure

The procedure was performed under sterile conditions in the operating room of the Eye and ENT Hospital of Fudan University in Shanghai, China. ATE-CXL was started with topical anesthesia using oxybuprocaine hydrochloride eye drops. After placing a lid speculum, the ParaCel solution (0.25% riboflavin and benzalkonium chloride, Avedro) was instilled for 4 min in the trephine (66 vision Tech, China) which was placed in central cornea. Then the cornea was soaked in the VibeX Xtra solution (0.25% riboflavin solution, Avedro) for 6 min. Subsequently, the cornea was rinsed with a balanced salt solution. Ultraviolet-A radiation with Avedro’s KXL System (Avedro, Inc) was given at an irradiance of 45 mW/cm^2^ in pulsed mode (1s on, 1s off) for 5 min and 20s, delivering a dose of 7.2 J/cm^2^. During irradiation, a balanced salt solution was applied to prevent the cornea from dehydration. After the irradiation, a bandage contact lens was patched on the eye, which was removed after 3 days. Postoperatively, patients received topical antibiotics (levofloxacin) and artificial tears (4 times per day for 1 week and 1 month, respectively). Then, 0.1% fluorometholone was prescribed seven times per day; the dose frequency was tapered gradually over 2 weeks.

### Data analysis

Statistical analysis was performed with SPSS. V23.0 (SPSS, Chicago, IL). Descriptive statistical data were displayed as mean ± standard deviation (SD) for continuous data. The variables were investigated using the Kolmogorov–Smirnov test to determine their normality and homogeneity. Each follow-up result was compared with the preoperative values, and the results were analyzed by the paired t-test, Wilcoxon rank-sum test, and repeated measures analyses of variance with Bonferroni-adjusted post-hoc comparisons. A logistic regression analysis was done at the last follow-up visit to predict the presence or absence of regression (decrease in Kmax values of ≥1 D), based on sex, age, and preoperative corneal astigmatism, K1, K2, Kmax, CCT, TCT, ACD, ACV, and cone position. The intergroup comparison of subgroups divided by Kmax and CCT were analyzed by Chi-square test. A *P*-value of < 0.05 was deemed statistically significant.

## Results

All operations were successfully completed without postoperative complications. No other adverse event including endoethelial damage was observed in any eye. ECD at baseline was 3047.20 ± 296.29 cells/mm^2^, and was 2996.48 ± 265.47 cells/mm^2^ at 12 months postoperatively (*P* = 0.152). No significant changes were found in BCVA (in logMAR units), which was 0.32 ± 0.15 preoperatively and 0.28 ± 0.13 at 1 year post-ATE-CXL (*P* = 0.085).

### Corneal topography outcomes

**Fig. 1:**
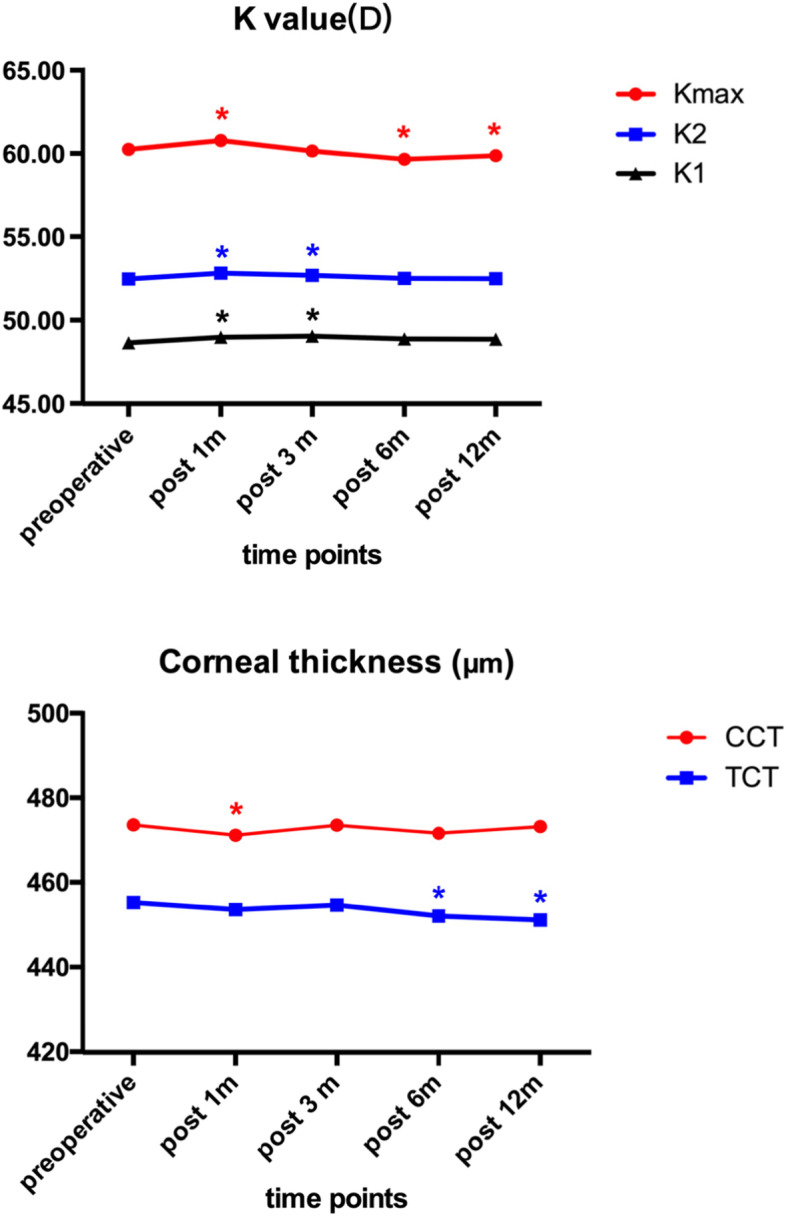
The Kmax, K1, K2, CCT and TCT values measured before and at 1, 3, 6 and 12 months after ATE-CXL. The asterisk indicates a significant difference (*P* < 0.05) that was detected when compared with the preoperative value. (Kmax, the maximum keratometry; K1, steepest meridian keratometry; K2, flattest meridian keratometry; CCT, central corneal thickness; TCT, thinnest corneal thickness; ATE-CXL, accelerated transepithelial corneal cross-linking)

The Kmax values were 60.25 ± 10.10 D preoperatively, and 60.79 ± 10.5 D, 60.15 ± 9.84 D, 59.65 ± 9.58 D, and 59.88 ± 9.92 D postoperatively at 1, 3, 6, and 12 months, respectively. Mean changes of Kmax from baseline at 1, 3, 6, and 12 months were 0.54 ± 1.51 D (*P* = 0.001), − 0.09 ± 1.46 D (*P* = 0.534), − 0.60 ± 2.21 D (*P* = 0.011) and − 0.36 ± 1.58 D (*P* = 0.030), respectively. Pre- and postoperative Kmax, K1, and K2 values are shown in Fig. 1.

The TCT values were 455.37 ± 43.55 μm preoperatively, and 453.71 ± 43.88 μm, 454.74 ± 46.76 μm, 452.19 ± 46.22 μm, and 451.22 ± 46.51 μm postoperatively at 1, 3, 6, and 12 months, respectively. Mean changes of TCT from baseline at 1, 3, 6, and 12 months were − 1.66 ± 11.90 μm (*P* = 0.183), − 0.62 ± 14.49 μm (*P* = 0.679), − 3.17 ± 12.94 μm (*P* = 0.020) and − 4.15 ± 15.78 μm (*P* = 0.013), respectively. The CCT values showed a reduction in pachymetry at 1 month postoperatively. Pre- and postoperative TCT and CCT values are presented in Fig. 1.

The corneal astigmatism decreased from 3.81 ± 2.58 D preoperatively to 3.63 ± 2.52 D and 3.60 ± 2.43 D at 3 and 6 months postoperatively (*P* = 0.049 and *P* = 0.048). Furthermore, the ACD values decreased from preoperatively to 12 months postoperatively (3.40 ± 0.27 vs 3.39 ± 0.25, *P* = 0.012).

Compared with baseline, PCE significantly decreased from 6.76 ± 0.74 μm preoperatively to 6.73 ± 0.74 μm at 1 month postoperatively (*P* = 0.004), whereas not significantly different at 3, 6 and 12 months following ATE-CXL. No significant changes were observed in PRC, PCE and PME at any time point during follow-up (*P* > 0.05) (Table 2).

**Table 2:** The ARC, PRC, PCE and PME values measured before and after ATE-CXL

	Preoperative	Post 1 m	Post 3 m	Post 6 m	Post 12 m
ARC (mm) *P* value	6.76 ± 0.74	6.73 ± 0.74 (*P* = 0.004)	6.73 ± 0.74 (*P* = 0.050)	6.75 ± 0.74 (*P* = 0.619)	6.74 ± 0.74 (*P* = 0.357)
PRC (mm) *P* value	5.26 ± 0.70	5.26 ± 0.71 (*P* = 0.283)	5.25 ± 0.71 (*P* = 0.237)	5.25 ± 0.70 (*P* = 0.277)	5.26 ± 0.71 (*P* = 0.744)
PCE (μm) *P* value	51.71 ± 34.12	52.57 ± 35.43 (*P* = 0.469)	52.78 ± 37.08 (*P* = 0.606)	53.32 ± 36.35 (*P* = 0.310)	52.98 ± 36.58 (*P* = 0.832)
PME (μm) *P* value	−18.25 ± 12.63	−18.32 ± 12.60 (*P* = 0.154)	−18.23 ± 12.59 (*P* = 0.716)	−18.15 ± 12.37 (*P* = 0.643)	−18.33 ± 12.61 (*P* = 0.797)

*ATE-CXL* accelerated transepithelial corneal cross-linking, *ARC* anterior radius of curvature, *PRC* posterior radius of curvature, *PCE* posterior central elevation, *PME* posterior mean elevation

### Factors affecting the keratoconus progression

To assess the factors affecting progression, logistic regression was used. It was found that the preoperative values of CCT and Kmax were predictors determining progression postoperatively (*P* = 0.003; *P* = 0.003). However, there was no significant correlation between other preoperative parameters and the regression existence at 12 months (Table 3).

**Table 3:** Logistic regression analysis between the regression existence at 12 months and preoperative parameters

Parameter	*P* value
PREOP Gender	0.677
PREOP Age	0.055
PREOP K1	0.291
PREOP K2	0.231
PREOP Kmax	0.003*
PREOP Corneal astigmatism	0.474
PREOP TCT	0.563
PREOP CCT	0.003*
PREOP ACD	0.373
PREOP ACV	0.964
PREOP Cone eccentricity	0.983
PREOP ARC	0.307
PREOP PRC	0.179
PREOP PCE	0.414
PREOP PME	0.216

Dependent factor: the regression existence at 12 months. *PREOP* preoperative; K1, steepest meridian keratometry, *K2* flattest meridian keratometry, *Kmax* maximum keratometry, *TCT* thinnest corneal thickness, *CCT* central corneal thickness, *ACD* anterior chamber depth, *ACV* anterior chamber volume, *ARC* anterior radius of curvature, *PRC* posterior radius of curvature, *PCE* posterior central elevation; *PME*, posterior mean elevation.

*P < 0.05

### Progress status after ATE-CXL

In all patients, the progress status at 12 months postoperatively was as follows: progressive (18.3%), stable (53.8%), and regressive (27.9%). Additionally, 14 (15.1%) treated eyes showed progression with increases of Kmax by > 1–2 D, 2 (2.2%) eyes showed increases of Kmax by > 2–3 D, and 1 (1.1%) treated eye showed an increase of Kmax by 3.2 D.

#### Grouping according to CCT

**Fig. 2:**
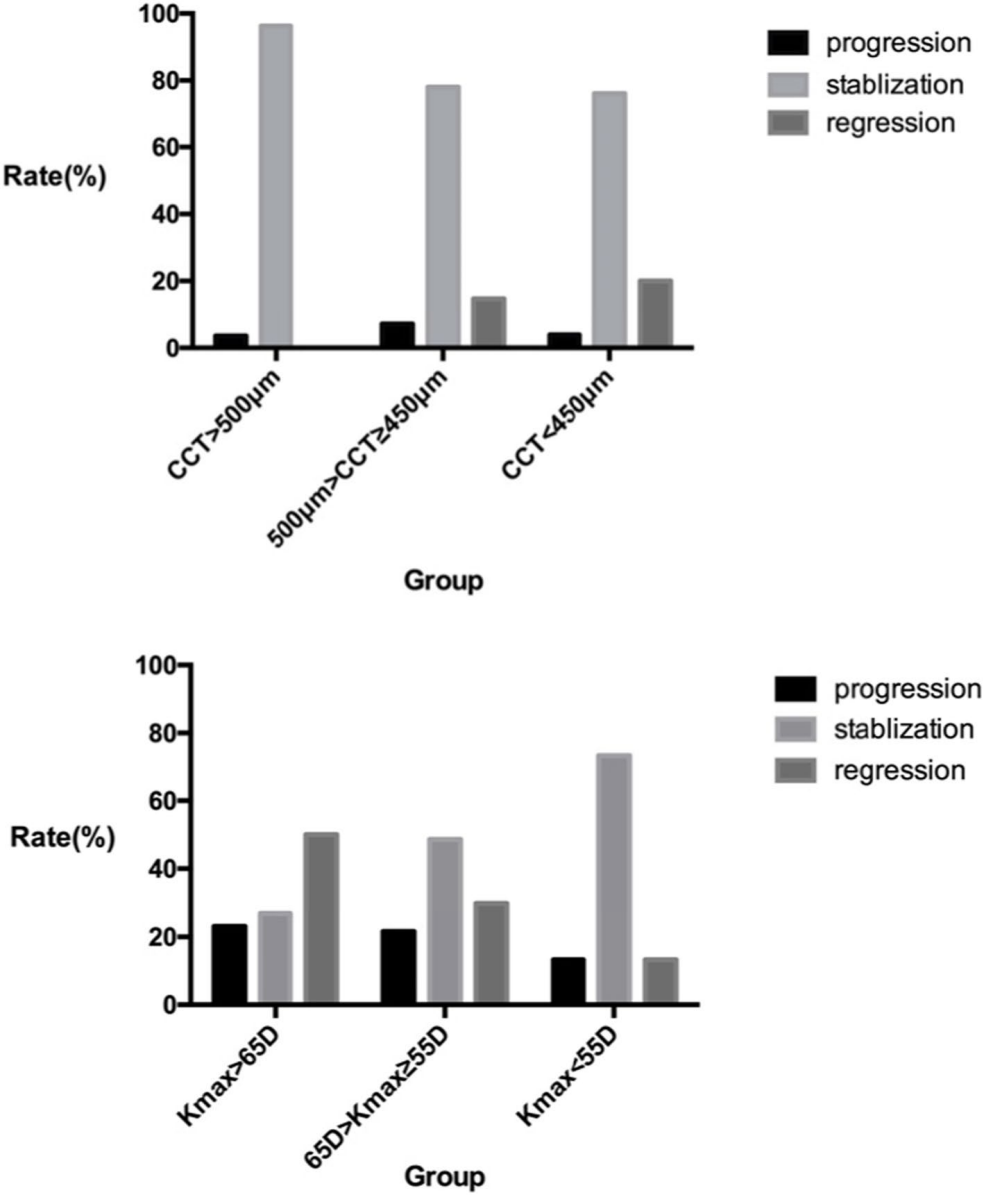
The rate of progress status of each group which grouped according to preoperative CCT and Kmax at 12 months after ATE-CXL. (CCT, central corneal thickness; Kmax, the maximum keratometry; ATE-CXL, accelerated transepithelial corneal cross-linking)

The stablization rates in group 1 (CCT > 500 μm), 2 (500 μm > CCT ≥ 450 μm) and 3 (CCT < 450 μm) were as follows: 96.3, 78.0 and 76.0%; The regression rates in three groups were as follows: 0, 14.7 and 20%. There were significant differences in status rates among three groups (*P* = 0.018). The progress status of each group is presented in Fig. 2.

The K2 values were increased at 1 month (*P* = 0.024) in group 1. In group 2, the ACV values were decreased at 3 months (*P* = 0.054); the Kmax values were increased at 1 month (*P* = 0.021); the TCT values decreased significantly from 454.85 ± 20.42 μm preoperatively to 449.68 ± 22.45 μm at 6 months postoperatively (*P* = 0.025). In group 3, the ACD values were significantly decreased from preoperatively to 12 months postoperatively (*P* = 0.008); the Kmax values showed a significant reduction at 6 and 12 months postoperatively (*P* = 0.018 and *P* = 0.045).

#### Grouping according to Kmax

The stablization rates in group 1 (Kmax > 65 D), 2 (65 D > Kmax ≥55 D) and 3 (Kmax < 55 D) were as follows: 26.9, 48.6 and 73.4%; The regression rates in three groups were as follows: 50.0, 29.8 and 13.3%. There were significant differences in status rates among three groups (*P* = 0.011). The progress status of each group is presented in Fig. 2.

In group 1, the ACD values showed significant decrease at 12 months (*P* = 0.039); the ACV values were significantly decreased at 3 and 6 months (*P* = 0.006 and *P* = 0.020); the Kmax values were increased at 1 month (73.03 ± 6.91 D vs 74.03 ± 7.48 D, *P* = 0.026), but showed a significant decrease at 6 months postoperatively (71.37 ± 7.09 D, *P* = 0.025). In group 2, the ACV values were decreased at 3 months (*P* = 0.054) postoperatively; no statistically significant (*P* > 0.05) changes were found for Kmax values during the 1-year follow-up. In group 3, the K2 and Kmax values significantly increased from preoperatively to 1 month postoperatively (*P* = 0.001, *P* = 0.047). The progress status of each group is presented in Fig. 2.

## Discussion

CXL is an effective treatment for halting keratoconus progression. It makes the corneal collagen links compactly through a photochemical reaction, by the use of riboflavin and ultraviolet-A radiation [2–6]. ATE-CXL, however, is a newer treatment, and the relationship between the preoperative parameters and clinical outcome should be evaluated.

In this study, the Kmax decreased by 0.60 ± 2.21 D and 0.36 ± 1.58 D at 6 and 12 months postoperatively, suggesting that ATE-CXL is an effective treatment for keratoconus and can halt its progression. Toprak et al. [21] reported that Kmax significantly decreased from 54.54 ± 5.50 D preoperatively to 53.52 ± 5.18 D at the 1-year follow-up of 96 eyes after C-CXL; similar results were observed by Greenstein et al. [22] and Badawi et al. [26] Our results were also in concordance with these studies. According to the previous literature, the keratoconus eyes had a significantly higher ACD than normal eyes [27], and this value increased with the severity of keratoconus [28]. Our study found that ACD decreased at 12 months postoperatively, also suggesting that ATE-CXL is an effective treatment for halting the progression of keratoconus.

We found that corneal astigmatism decreased at 3 months and 6 months postoperatively, respectively. Badawi et al. [29] reported that corneal astigmatism decreased significantly in adolescent keratoconus after C-CXL during the 1-year follow-up. However, there was no significant difference in corneal astigmatism at 12 months postoperatively compared with the preoperative result in our study. The difference may be related to the age of the patient, as the corneal morphology of teenagers is easier to change. In addition, Hashemi et al. [30] found no significant difference in astigmatism at 4 years after C-CXL, suggesting that the alteration after C-CXL should be observed for a longer time. In this study, ARC, PRC, PCE and PME did not show significant changes at postoperative 12 months compared with the baseline, indicating that ATE-CXL may have prevented or delayed the progress of corneal ectasia and maintained structural stability of the cornea at 1 year after treatment.

Our results showed that CCT decreased at 1 month postoperatively, but there was no significant difference observed at 3, 6, and 12 months follow-up. There was an initial sharp decrease in corneal thickness possibly due to a decrease in the interlamellar space and compaction of stroma. However, after a period of time, the corneal thickness increased again due to the remodeling of collagen fibers [31]. Our study also found that TCT decreased by 3.17 ± 12.94 μm and 4.15 ± 15.78 μm at 6 and 12 months postoperatively. A previous study reported that TCT decreased significantly from 460.11 ± 47.15 μm to 430.65 ± 61.90 μm during the 1-year follow-up after C-CXL [21]. Ozer et al. [24] found that TCT decreased significantly by 21.7 ± 33.6 μm at 1 year after C-CXL. Similar results were observed by Sarac et al. [23], where TCT decreased significantly from 430.2 ± 54.4 μm to 411.5 ± 62.6 μm 2 years after C-CXL. Compared with the results of these studies, our results showed less decrease in TCT. The possible reason is that the epithelium is removed during C-CXL, while it is preserved in ATE-CXL; therefore, although TCT decreased significantly in this study, the decrease was lesser than that in previous studies of C-CXL.

In this study, we could not disregard 18.3% of treated eyes showed a tendency for progression at 1 year after ATE-CXL, which was a relatively big proportion comparing to those who were treated by C-CXL in some studies. The main reason might be the severe keratoconus accounted for a high proportion in this study (55.9% patients with Kmax greater than 58 D), so that the probability of progression was relatively higher. Similarly, in Kuechler et al’s study [32], the incidence of progression at 1 year after C-CXL was 23% in treating keratoconus eyes with Kmax values ≥58 D. However, compared with C-CXL, the ATE-CXL is more safe, noninvasive and makes patient more comfortable.

Here, the preoperative parameters and the changes of Kmax were analyzed by logistic regression analysis. It was found that the thinner preoperative CCT or greater Kmax values have greater probability of flattening ≥1.0 D in Kmax values after ATE-CXL, which indicates that ATE-CXL is an effective treatment for severe keratoconus. Ozer et al. [24] found that the change in Kmax after C-CXL was related to CCT in 4-year-follow-up. In contrast to our study, Toprak et al. [21] found that Kmax values alteration were related to age. Here, the age of the study population was < 30 years (84.9% of all cases), that might have affected statistics. Godefrooij et al. [20] and Wisse et al. [19], reported that the change of Kmax values was related to the cone eccentricity after C-CXL, but here, the preoperative cone position of most patients was within 3 mm (82.8% of all cases), which could have affected the results.

In order to observe the effect after ATE-CXL under different preoperative conditions, we grouped the patients according to CCT and Kmax values before operation. We found that the probability of flattening (Kmax decrease of ≥1.0 D) gradually increased from 0 to 20% with the decrease of CCT thickness. The more serious keratoconus has thinner cornea, and an increase in infiltration depth of riboflavin leads to deeper effect in cornea during CXL. Therefore, the thicker cornea has the shallower depth of infiltration in stoma, and the effect of CXL may be affected. Toprak et al. [21] reported that patients with TCT < 450 μm suggested a decrease in Kmax after C-CXL, whereas Sarac et al. [23] reported an ease in progression after CXL, as thinner cornea represents more severe keratoconus, making the possibility of progress greater. The contradictory results suggest that the effects of CXL with thinner cornea should be confirmed by more cases. Zhang observed that the patients with TCT less than 450 μm had the greater decreasing in average keratometry after ATE-CXL [17] in 42 eyes from 37 patients, which was similar to our results.

Our results also showed that the probability of flattening (Kmax reduction of ≥1.0 D) gradually decreased from 50 to 13.3% with the decrease of Kmax, suggesting that the patients with greater preoperative Kmax values have a better effect after ATE-CXL, which could be partly attributable to the relatively deep cross-linking in eyes with advanced keratoconus. However, our results also showed that the progressive rate increased with the rise of Kmax, and the reason might be the natural progression rate of keratoconic eyes in advanced subgroup are more likely higher than in mild subgroup. The study by Koc et al. [25] showed similar results in the 1-year follow-up after C-CXL. Greenstein et al. [22] reported that the patients with a Kmax value of ≥55.0 D, were more likely to improve by decreasing more than 2.0 D in Kmax. According to Koller et al. [33], patients with a Kmax value of ≥54.0 D had a greater decrease in Kmax. These studies suggested that the preoperative Kmax values had an impact on the Kmax values after CXL; however, the specific relationship needs to be further explored.

There are some limitations in our study. The follow-up time was only 1 year, which can be increased to obtain the long-term clinical effects of the treatment. In addition, there are some published studies that have assessed the corneal biomechanical properties of patients with keratoconus by Corvis-ST, which can be added in the follow-up examination to evaluate the safety of ATE-CXL.

Our study suggests that ATE-CXL is a safe and effective treatment for patients with keratoconus. Patients with thinner CCT and higher Kmax values were most likely to improve after ATE-CXL, but the long-term effects are yet to be elucidated and need further observation.

## Data Availability

The datasets used and analyzed during the current study are available from the corresponding author on reasonable request.
